# CAFs affect the proliferation and treatment response of head and neck cancer spheroids during co-culturing in a unique in vitro model

**DOI:** 10.1186/s12935-020-01718-6

**Published:** 2020-12-22

**Authors:** Mustafa Magan, Emilia Wiechec, Karin Roberg

**Affiliations:** 1 Division of Cell Biology, Department of Biomedical and Clinical Sciences, Linköping University, Linköping, Sweden; 2 Department of Otorhinolaryngology in Linköping, Anesthetics, Operations and Specialty Surgery Center, Region Östergötland, Linköping, Sweden

**Keywords:** Head and neck squamous cell carcinoma, Tumor spheroids, Cancer associated fibroblasts, Cancer stem cells, Epithelial–mesenchymal transition, Drug response

## Abstract

**Background:**

Head and neck squamous cell carcinoma (HNSCC) is a heterogeneous group of tumors for which the overall survival rate worldwide is around 60%. The tumor microenvironment, including cancer-associated fibroblasts (CAFs), is believed to affect the treatment response and migration of HNSCC. The aim of this study was to create a biologically relevant HNSCC in vitro model consisting of both tumor cells and CAFs cultured in 3D to establish predictive biomarkers for treatment response, as well as to investigate the impact of CAFs on phenotype, proliferation and treatment response in HNSCC cells.

**Methods:**

Three different HNSCC patient-derived tumor cell lines were cultured with and without CAFs in a 3D model. Immunohistochemistry of the proliferation marker Ki67, epidermal growth factor receptor (EGFR) and fibronectin and a TUNEL-assay were performed to analyze the effect of CAFs on both tumor cell proliferation and response to cisplatin and cetuximab treatment in tumor spheroids (3D). mRNA expression of epithelial-mesenchymal transition (EMT) and cancer stem cells markers were analyzed using qRT-PCR.

**Results:**

The results demonstrated increased cell proliferation within the tumor spheroids in the presence of CAFs, correlating with increased expression of EGFR. In spheroids with increased expression of EGFR, a potentiated response to cetuximab treatment was observed. Surprisingly, an increase in Ki67 expressing tumor cells were observed in spheroids treated with cisplatin for 3 days, correlating with increased expression of EGFR. Furthermore, tumor cells co-cultured with CAFs presented an increased EMT phenotype compared to tumor cells cultured alone in 3D.

**Conclusion:**

Taken together, our results reveal increased cell proliferation and elevated expression of EGFR in HNSCC tumor spheroids in the presence of CAFs. These results, together with the altered EMT phenotype, may influence the response to cetuximab or cisplatin treatment.

## Background

Head and neck cancer is a collective term for cancers that arise in the lips, oral cavity, salivary glands, paranasal sinuses (including middle ear cancer), pharynx, larynx and trachea. More than 90% of all head and neck cancers are squamous cell carcinoma (HNSCC), originating from the epithelial cell layer. These epithelial tumors are caused by multiple genetic alterations and are the seventh most common type of cancer worldwide [1]. Head and neck cancer treatments often require different combinations of chemotherapy (mostly including cisplatin), surgery and radiotherapy, but treatment resistance and local recurrence are still significant problems [1]. Furthermore, in recent years inhibition of EGFR signaling with the monoclonal EGFR antibody cetuximab (Erbitux®, Merck KGaA) has emerged as a new treatment strategy. To significantly increase overall survival in HNSCC patients, personalized targeted therapy is required, which depends on the establishment of novel biomarkers that can predict treatment response.

The most common method of investigating tumor cells in vitro is to use two (2D) dimensional monolayer cell cultures. However, 2D cell cultures lack most of the cell–cell interactions seen in 3D and therefore preclude several physiological processes present in solid tumors, making 2D cultures poor predictors of clinical benefit [2, 3]. Different studies have indicated that spheroid cell culture (3D, three-dimensional cell growth) is superior to 2D culture with respect to mimicking tumor environment owing to cell–cell communication, cell–matrix interconnections and along investigation of cell morphology, proliferation, differentiation and signal transduction. Therefore, spheroid cell cultures in various studies have been shown to reflect cancer biology more accurately in vitro [2, 3]*.* Decreased sensitivity to cytotoxic agents (doxorubicin, cisplatin and 5-fluorouracil) has been shown in tumor cells cultured as spheroids compared to 2D cultures [4, 5]. In a previous study, we showed that most investigated HNSCC cell lines exhibited decreased sensitivity to cisplatin and cetuximab when cultured in 3D compared to 2D. Moreover, increased expression of cancer stem cell-associated genes (Nanog and Sox2) and E-cadherin were observed in all investigated cell lines in 3D, as well as decreased proliferation [6].

The most abundant stromal cell type in epithelial tumors are cancer-associated fibroblasts (CAFs) [7]. CAFs have been shown to play a significant role in facilitating tumor progression and are associated with poor prognosis in many different types of cancer, including HNSCC [8]. Varying levels of autocrine and paracrine cytokines, as well as other tumor promoting factors, are secreted by CAFs to facilitate tumor proliferation, angiogenesis, invasion, immune escape, drug resistance and metastasis [8, 9].

The morphological and functional characteristics of CAFs are very different from normal fibroblasts. It is believed that HNSCC cells secrete soluble tumor factors (ex TGF-1β, TNF-α, IL-6) that induce differentiation of normal fibroblasts into CAFs, which are constantly activated [8, 10, 11]. Moreover, reciprocal communication between CAFs and tumor cells is required, where CAFs, in turn, upregulate tumor stimulating mediators (vimentin, matrix metalloproteinases, periostin, IGF2, BDNF, IL-33, and CXCL12), which are important for tumor growth, invasion and downregulation of tumor suppressor genes, like p53 and p21 [8, 9, 11]. In HNSCC, it has been shown that CAFs from HNSCC tumors co-cultured in 2D with HNSCC cell lines increase tumor cell proliferation and decrease sensitivity to cetuximab [12]. Similarly, others have shown that co-culturing CAFs with tumor cells in 3D increases the proliferation of pancreatic, breast and lung cancer cells [13].

The aim of this study was to create a biologically relevant HNSCC in vitro model consisting of both tumor cells and CAFs cultured in 3D that could be valuable in the search for predictive biomarkers for treatment response. We used HNSCC cells and CAFs established from the same pre-treatment biopsies to investigate the impact of CAFs on proliferation and treatment response. Additionally, the phenotype of tumor cells/CAFs spheroids was compared to the tumor phenotype.

## Material and methods

### Tumor samples and cell lines

Samples from patients with HNSCC were obtained from an established tumor collection (No 416, The National Board of Health and Welfare in Sweden) at the Department of Otorhinolaryngology, Head and Neck Surgery, at the University Hospital of Linköping, Sweden (approved by the Ethical Committee of Linköping). Tumor and CAFs cell lines were established from one part of pre-treatment biopsies [14], and the other part was formalin-fixed and paraffin-embedded.

### Cell culture conditions

Three HNSCC cell lines, LK0902, LK0917 and LK1108 from the Linköping University collection, were used in this study (Table 1). All cell lines were derived from tissue specimens from patients diagnosed with HNSCC and were cultured in Keratinocyte-SFM supplemented with antibiotics (penicillin 50 U/ml, streptomycin 50 µg/ml) and 10% FBS (all from GIBCO, Invitrogen Corporation, Paisly, UK)[14]. The cells were given fresh culture media twice per week and were subcultured at confluence after detaching the cells with 0.25% trypsin + 0.02% EDTA (GIBCO) at a weekly split ratio of approximately 1:2 or 1:3. Cultures in passages 10 to 25 were used for all experiments. Mutations in the p53 gene in the tumor biopsy and the tumor-derived cell line were used to confirm identity of tumor cells.

**Table 1:** Origin and tumor characteristics of the investigated cell lines

Cell line	Primary tumor location	TNM	Gender
LK0902	Tongue	T1N0M0	F
LK0917	Gingiva	T4N1M1	M
LK1108	Hypopharynx	T2N0M0	F

TNM classification according to the International Union against Cancer (UICC, 2002)

CAFs were established at the same time as the tumor cell lines and were cultured under the same conditions. CAFs were identified due to their morphology and their expression of vimentin as earlier described [12]. The cells were screened periodically for mycoplasma contamination using the MycoAlert Mycoplasma Detection Kit (Lonza, Walkersville, USA).

### Tumor spheroids

To achieve spheroids of tumor cells or co-cultures of tumor cells and CAFs, 15,000 tumor cells or 10,000 tumor cells with 5000 CAFs were seeded into each well of ultra-low attachment (ULA) 96-well round-bottomed plates (Corning, Amsterdam, The Netherlands) and were thereafter cultured in standard culture conditions.

For treatment, spheroids were cultured for 48 h before addition of cetuximab (60 nM; Erbitux®, Merck KGaA, Darmstadt, Germany) or cisplatin (4 µg/ml; Cisplatin Meda, Sandoz A/S, Denmark) to selected spheroids in triplicate for 3 days. Untreated spheroids were used as controls for each treatment.

### Immunohistochemistry (IHC) and TUNEL-assay

Spheroids were fixed in 4% paraformaldehyde (Santa Cruz Biotechnology, Dallas, TX, USA) over night at 4 °C followed by rinsing with PBS, stained with 0.1% toluidine blue D (Merck, Kenilworth, NJ, USA) and centrifuged in 2% agarose. Agarose blocks containing spheroids were dehydrated using an accelerating ethanol series followed embedding in paraffin wax (Merck, Kenilworth, NJ, USA). Sections of 5 μm were collected on Super Frost Plus slides (Thermo Fisher Scientific, Fremont, CA, USA), dried overnight and then incubated at 58 °C for 1 h. Sections were deparaffinized in Histolab-Clear (HistoLab, Gothenburg, Sweden), re-hydrated in graded alcohols, underwent antigen retrieval (PT 200, DAKO Denmark A/S) and were then blocked for endogenous peroxidase activity in 3% peroxidase (Sigma-Aldrich, St. Louis, MO, USA). After incubation in blocking buffer (0.1% BSA-5% FBS in TBS-Triton), sections were incubated overnight at 4 °C with primary antibodies: Ki-67 (1:50; Santa Cruz Biotechnology), EGFR (1:50; Cell Signaling Technology, Danvers, MA, USA) or fibronectin (1:200, Sigma-Aldrich). Thereafter, sections were washed in TBS-triton buffer and incubated with a goat anti-rabbit secondary antibody (1:500; IgG, EMD Millipore Corporation, Temecula, CA, USA) for 60 min at room temperature. Following the washing steps, slides were developed for 4 min using the ImmPACT NovaRED peroxidase substrate kit at room temperature (Vector Laboratories, Burlingame, CA, USA). Thereafter, sections were counterstained in Mayer´s hematoxylin (HistoLab) for 4 min, dehydrated, cleared in Histolab-Clear and cover slipped with Pertex (HistoLab). As negative controls sections were stained as above described but without the primary antibody. All negative controls were revealed unstained (data not shown).

Apoptotic cells were detected in sections from the paraffin embedded tumor spheroids (see above) using a TUNEL assay by means of the HRP-DAB TUNEL staining kit (ab206386, Abcam, Cambridge, UK) according to the manufacturer’s instructions. Methyl green was used to counterstain the slides. Images were acquired with a light microscope (Zeiss LSM 700; Zeiss, Oberkochen, Germany) with a 20× objective.

### Image analysis of Ki67 positive cells

The extent of Ki67 positive cells was analyzed in at least 6 images from 2 experiments. Positive cells were counted in all spheroids or part of spheroids in each image. The measurement of chosen areas containing tumor spheroids was performed using ImageJ 1.47 v software (National Institutes of Health, Bethesda, MD). Briefly, single spheroids within the analyzed area were marked manually with polygon selection tool. Next, the pixel values of single spheroids analyzed by ImageJ were imported to Excel where the mean area and standard deviation were calculated. The results were presented as Ki67+ cells/area.

### Magnetic activated cell sorting

Tumor cells in spheroids were separated from CAFs by magnetic activated cell sorting (MACS). Spheroids were sampled in a test tube, centrifuged (1000 rpm, 5 min), washed in PBS, centrifuged and then incubated with 500 µl trypsin for 5 to 20 min at 37 °C. Thereafter, 1500 µl culture media was added, and the cells were flushed 30 times with a micropipette and then subjected to separation. The tumor cells or the tumor cells/CAFs cell suspensions were magnetically labelled with Anti-fibroblast Micro Beads (Miltenyi Biotech Norden, Lund, Sweden) by a 15-min incubation at 4 °C. The cells were washed, suspended in PBS and passed through a 50 µm filter (Becton Dickinson, USA). The cell suspensions were then loaded onto MACS® Column Beads (Miltenyi Biotech). The tumor cells were collected in test tubes placed under the column, and the CAFs were collected in the column due to the magnetic field. This procedure was repeated three times with each suspension to increase the purity of each population. To check the purity of the tumor cell suspensions, cells were cultured for 48 h and thereafter 500 cells with epithelial or fibroblast morphology were counted in a phase contrast light microscope. Less than 1% of the cells were found with a fibroblast morphology in all samples.

### RT-qPCR

The RT-qPCR analysis was performed on a 7500 Fast Real-Time PCR system (Applied Biosystems, Waltham, MA, USA). Total RNA was extracted from cells using the RNeasy Mini Kit (Qiagen, Hilden, Germany), cDNA was synthesized using the High Capacity RNA-to-cDNA Kit (Applied Biosystems), and TaqMan FAM/MGB probes (Applied Biosystems) were used for PCR reactions. Amplification of both glyceraldehyde 3-phosphate dehydrogenase (GAPDH) and ß-actin was used as an internal standard. The comparative Ct method was applied to determine the fold-difference in expression levels relative to a control sample [15].

### Assessment of viability after cisplatin treatment

Tumor cells were seeded into 96-well ULA plates and cultured for 48 h before exposure to cisplatin (4 µg/ml; Cisplatin Meda, Sandoz A/S, Denmark). Cisplatin was added to selected spheroids, and cytostatic/cytotoxic effects were assessed after another three days of culture. Eight spheroids were sampled in an Eppendorf test tube, centrifuged (1000 rpm, 5 min), washed in PBS, centrifuged again, and then incubated with 200 µl trypsin for 5 to 20 min at 37 °C. Thereafter, 200 µl culture media was added, and the cells were flushed 30 times with a micropipette and then subjected to MTS assay.

The viability of treated tumor cells was measured using the CellTiter 96® AQ_ueous_ One Solution Cell Proliferation Assay (Promega, Madison, WI, USA). Briefly, treated and untreated tumor cells grown in 3D (dissociated spheroid-derived cells) were added to a 96 well plate and 20 µl/100 µl medium of the MTS substrate was then added followed by a 3-h incubation at 37 °C. The absorbance of each well was measured at λ = 490 nm with a VersaMax (Molecular Devices, San Lose, CA, USA) microplate reader. All analyses were performed three times, and the mean values were used for further calculations.

### Statistics

The data are presented as the mean ± SD. All experiments were repeated at least in triplicate. The data were analyzed using one-way ANOVA with Bonferroni adjustment using GraphPad Prism software (GraphPad Software, San Diego, CA, USA). P-values ≤ 0.05 were considered significant.

## Results

### CAFs increase the cell proliferation in tumor spheroids

**Fig. 1:**
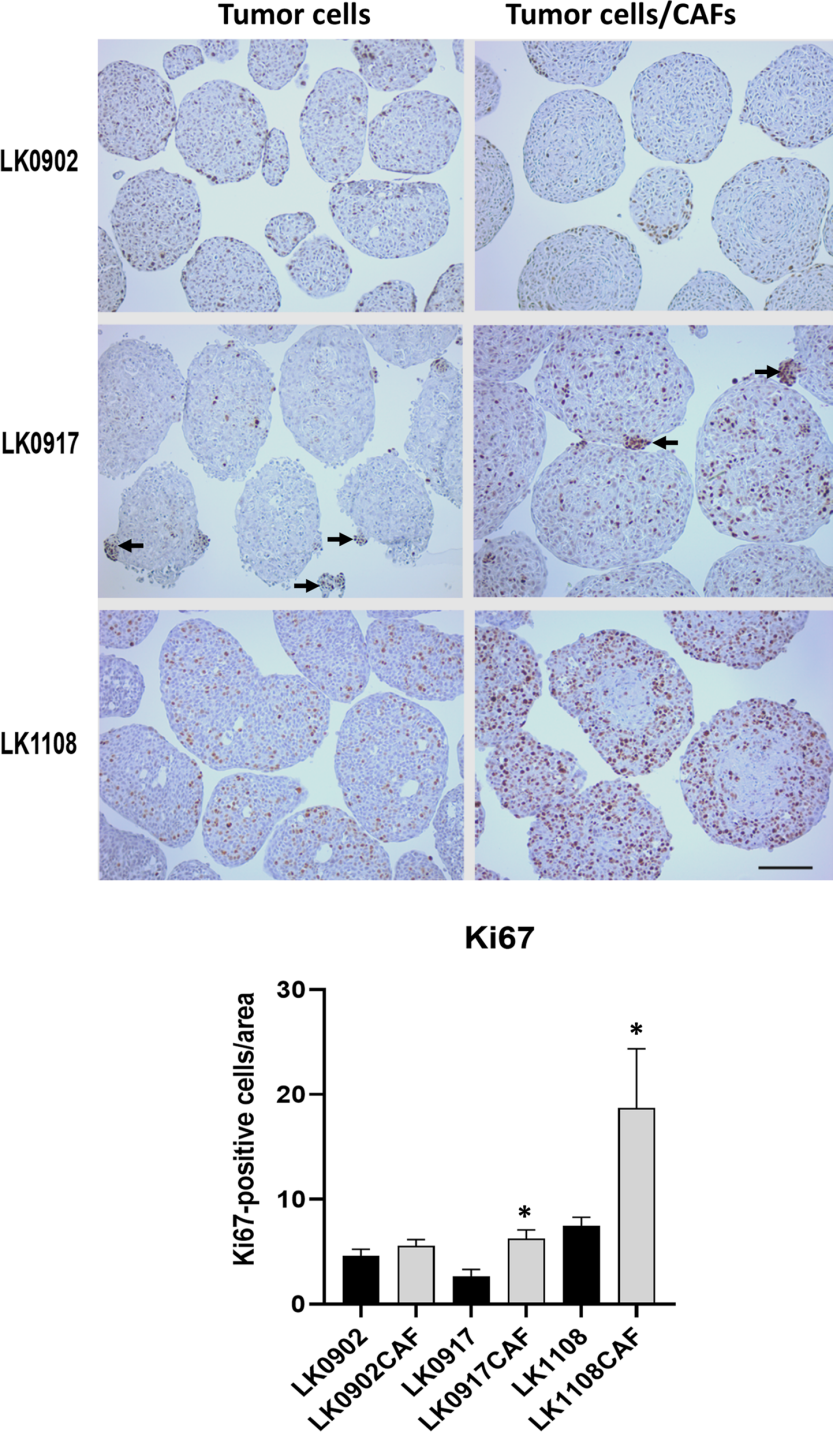
Ki67 expression in HNSCC cells grown in 3D ± CAFs. Immunohistochemical staining of untreated HNSCC tumor spheroids ± CAFs with the proliferation marker Ki67 cultured for 5 days: scale bar = 150 µm. Clusters with tumor cells are indicated with arrows. Quantification of Ki67-positive cells in 5 days old tumor spheroids; data are shown as Ki67 positive cells/area ± SD, 2 experiments, images = n ≥ 6. *p < 0.05 according to one-way ANOVA with Bonferroni adjustment

To investigate how CAFs affect the proliferation of tumor cells growing as spheroids, the tumor cell lines LK0902, LK0917 and LK1108 were cultured with and without CAFs as spheroids for 5 days. The expression of the proliferation marker Ki-67 was analyzed in paraffin sections by immunohistochemistry (IHC). The number of Ki67 positive cells was counted in all spheroids in each image and areas of the spheroids were measured using Image J software. Results are presented as mean Ki67-positive cells/area. Significantly increased numbers of Ki-67 positive cells were observed in LK0917 and LK1108 tumor cells/CAFs spheroids compared to tumor spheroids without CAFs (Fig. 1). Interestingly, in LK0917 spheroids, most Ki67 positive cells were found in small clusters of cells both inside the spheroids and detached as separated, smaller spheroids (indicated with arrows). Furthermore, clusters with Ki67 positive cells were also observed in tumor cells with CAFs spheroids but to a lesser extent (Fig. 1).

### CAFs affect treatment response

**Fig. 2:**
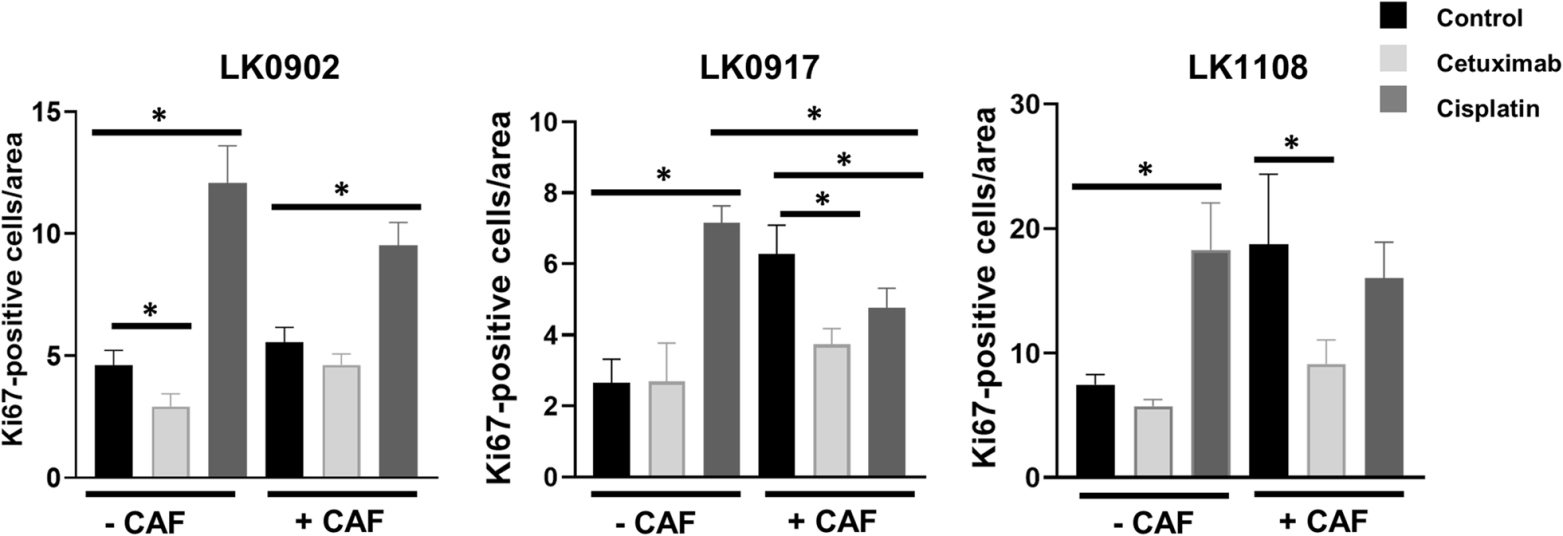
Ki67 expression in HNSCC cells grown in 3D ± CAFs after treatment with cisplatin and cetuximab. Proliferation in response to treatment with cetuximab and cisplatin was measured by immunohistochemical staining with the proliferation marker Ki67. Quantification of Ki67-positive cells in 5 days old tumor spheroids; data are depicted as Ki67 positive cells/area ± SD, 2 experiments, images = n ≥ 6. *p < 0.05 according to one-way ANOVA with Bonferroni adjustment

The impact of CAFs on treatment response was investigated in spheroids from LK0902, LK0917 and LK1108 HNSCC cell lines. Tumor cells were cultured with and without CAFs in spheroids for two days and thereafter treated with cetuximab or cisplatin for another three days. To analyze the treatment response, cell proliferation was assessed with Ki67 staining. The number of Ki67 positive cells was analyzed as described above and is presented as mean value of Ki67-positive cells/area. After cetuximab treatment, increased proliferation was observed in LK0902, LK0917 and LK1108 tumor cells/CAFs spheroids compared to spheroids with only tumor cells. However, when these spheroids were compared to untreated controls with CAFs, a significantly increased response to cetuximab was seen in LK0917 and LK1108 cell lines (Fig. 2, Additional file 1: Figure S1, Additional file 2: Figure S2 and Additional file 3: Figure S3).

Surprisingly, in the majority of cisplatin-treated spheroids with or without CAFs, an increase in number of Ki67 expressing cells was detected compared to untreated spheroids containing only tumor cells. However, in LK0917 tumor cells/CAFs spheroids, a significant decrease in Ki67 positive cells was detected after cisplatin treatment compared to spheroids with only tumor cells or compared to untreated tumor cells/CAFs spheroids (Fig. 2, Additional file 2: Figure S2). In a previous study, we showed that spheroids from LK0902 and LK0917 exhibited a significant decrease in cell viability (MTS assay) after seven days of cisplatin treatment [6]. Therefore, we analyzed cell viability after three days of cisplatin treatment in tumor spheroids from all three cell lines, and the results showed a significant decrease in cell viability in LK0917 spheroids in response to cisplatin treatment (Additional file 4: Figure S4).

To further investigate the treatment response, the number of apoptotic cells in spheroids were evaluated using a TUNEL-assay. In LK0902 and LK1108 tumor cells and tumor cells/CAFs spheroids only a small number of apoptotic cells were found. Moreover, after treatment with cetuximab and cisplatin only a limited increase of apoptosis was found (Additional file 1: Figure S1 and Additional file 3: Figure S3). In LK0917 tumor cells and tumor cells/CAFs untreated spheroids a number of apoptotic cells were noticed, and after treatment an increased apoptosis was found in tumor cells spheroids after cisplatin treatment and in tumor cells/CAFs spheroids after both cetuximab and cisplatin treatment (Additional file 2: Figure S2).

### CAFs increase the expression of EGFR

**Fig. 3:**
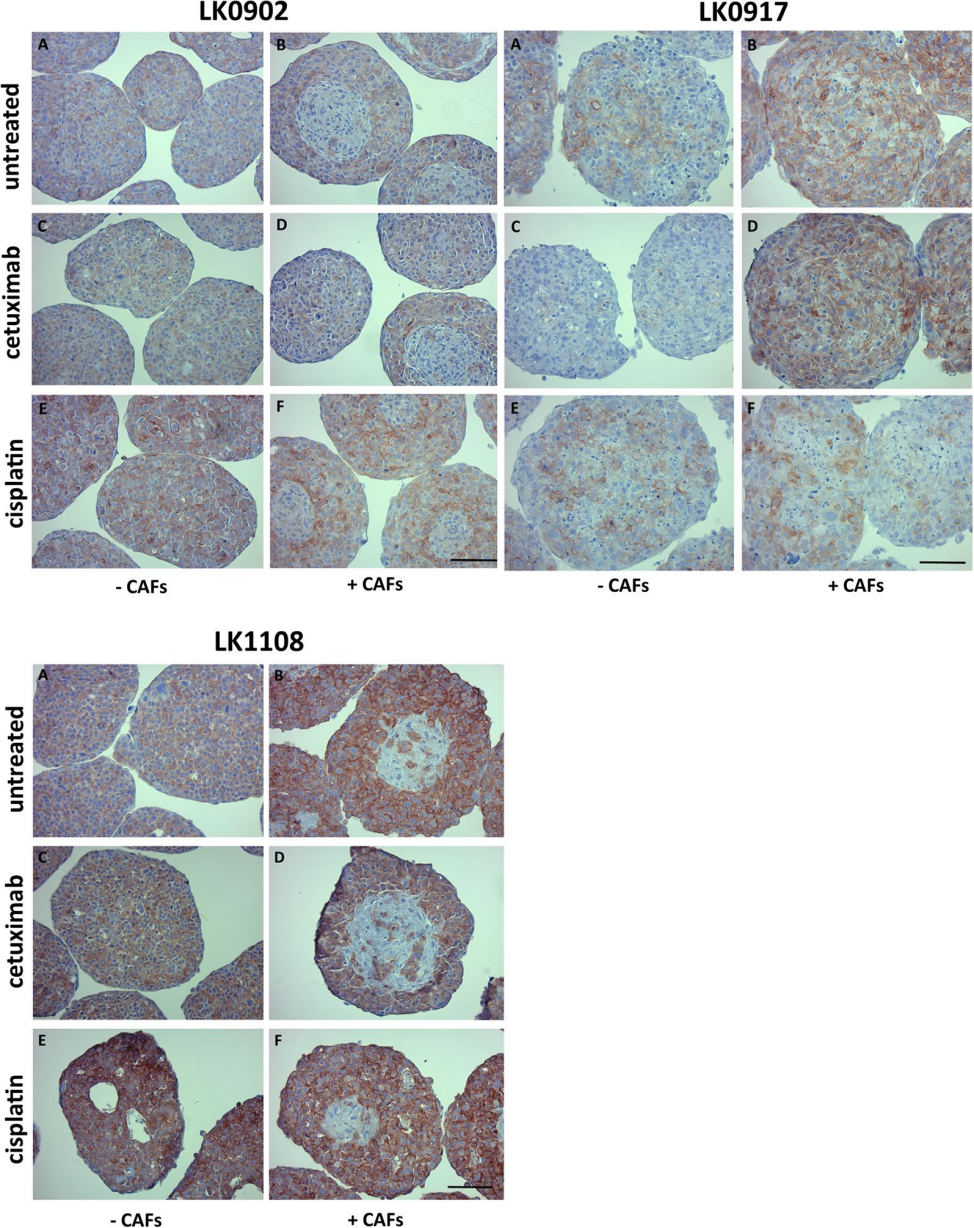
EGFR expression in HNSCC cells grown in 3D ± CAFs after treatment with cisplatin and cetuximab. Expression of epidermal growth factor receptor (EGFR) in response to treatment with cetuximab and cisplatin was measured in 5 days old tumor spheroids by immunohistochemical staining. Untreated spheroids (**a**, **b**). Cetuximab treated spheroids (**c**, **d**). Cisplatin treated spheroids (E, F). Scale bar = 150 µm

To determine whether CAFs affect EGFR expression in tumor cells, HNSCC cell lines were cultured with and without CAFs as spheroids for two days and thereafter treated with cetuximab and cisplatin for another three days. In all cell lines, increased expression of EGFR (IHC) was shown in tumor cells co-cultured with CAFs compared to spheroids composed solely of tumor cells. CAFs were not stained for EGFR and were mostly located in the central area of LK0902 and LK1108 spheroids, whereas the pattern of CAFs in LK0917 spheroids was more scattered (Fig. 3). Moreover, CAFs (EGFR negative cells) were positively stained for fibronectin (Additional file 5: Figure S5). Next, we compared expression of EGFR in pre-treatment tumor biopsies to expression in 3D spheroids composed of tumor cells and CAFs (established from the same biopsy). The LK0902 biopsy and spheroids exhibited the lowest expression of EGFR, and the highest EGFR expression was observed in the LK1108 biopsy and spheroids (Additional file 6: Figure S6).

After cetuximab treatment, only small changes in EGFR expression were observed. Surprisingly, after cisplatin treatment, an increase in EGFR expression was detected in all analyzed spheroids solely comprising tumor cells. In contrast, in spheroids with CAFs treated with cisplatin, increased EGFR expression was observed compared to untreated spheroids in LK0902 cells (Fig. 3). These results correlate with the proliferation rate as shown as Ki67 positive tumor cells (Fig. 2).

### CAFs induce changes in EMT and CSC phenotype

**Fig. 4:**
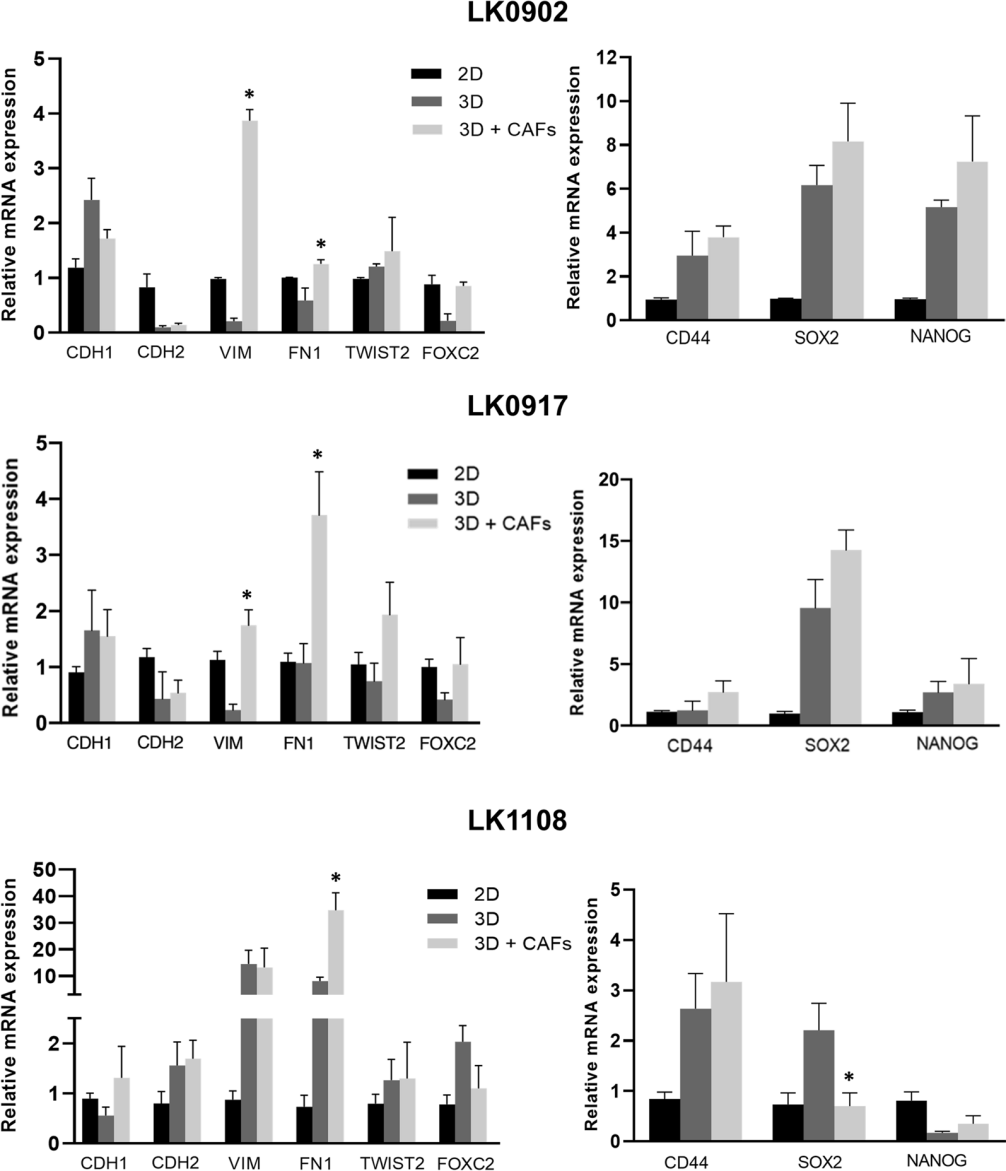
mRNA expression of EMT and CSC markers in 5 days old HNSCC tumor spheroids ± CAFs relative to cells cultured in 2D. Relative levels of analyzed genes were calculated using the 2 − ΔΔCt method, and GAPDH and β-actin were used as internal standards. Data are normalized to cells cultured in 2D; n = 3 experiments, *p < 0.05 according to one-way ANOVA with Bonferroni adjustment

The EMT and CSC phenotype of HNSCC cells grown in spheroids with and without CAFs was assessed by RT-qPCR. In all three cell lines, significantly increased mRNA expression of mesenchymal marker FN1 was detected in tumor cells sorted from co-culture of tumor cells and CAFs as spheroids compared to tumor cells grown as spheroids without CAFs. Furthermore, in LK0902 and LK0917 cells, increased VIM mRNA expression was observed in tumor cells from tumor/CAF spheroids (Fig. 4).

In our previous study we have shown that sphere culture conditions caused a significant increase in mRNA expression of NANOG and SOX2 compared to 2D cultures from all investigated HNSCC cell lines [6]. In this study, decreased mRNA expression of SOX2 was detected in LK1108 tumor cells sorted from co-culture of tumor cells and CAFs as spheroids when compared to tumor cells grown in spheroids alone. On the other hand, in LK0902 and LK0917 tumor cells from tumor cell/CAF spheroids, a tendency to an increase in mRNA expression of NANOG and SOX2 was observed (Fig. 4).

## Discussion

In the present study, a new and unique biologically relevant in vitro model was established. In this model, tumor cells and CAFs established from the same tumor biopsy were co-cultured in spheroids (3D). The results showed a similar distribution of CAFs in the spheroids as in the tumor biopsy, as well as similar EGFR expression (Fig. 3, Additional file 6: Figure S6). To our knowledge, there are no other experimental in vitro models consisting of low passage HNSCC cells and CAFs originating from the same tumor.

To establish an in vitro model with properties as close as possible to the tumor derived from patients that could be used for drug testing, as well as for development of new predictive markers, we cultured CAFs and tumor cells as 3D spheroids. The impact of CAFs, one of the abundant factors in the extracellular matrix, on proliferation and treatment response of HNSCC tumor cells in spheroids was investigated. The results revealed increased cell proliferation in the presence of CAFs, as evidenced by increased expression of Ki-67 in the spheroids compared to culture of these cell lines without CAFs. In a previous study, we demonstrated that both CAFs and conditioned medium from CAFs cultures stimulated increased proliferation of HNSCC cells [12]. Others have shown that CAFs promote tumor growth and progression of prostate tumors [16] and increase the proliferation of pancreatic, breast and lung tumor cells when co-cultured in spheroids [13]. Of note, in LK0917 cultures, most Ki67 expressing cells were found in clusters inside the spheroids, at the edge of the spheroids or in small, separated spheroids (Additional file 2: Figure S2, arrows). Furthermore, these Ki67 expressing cells exhibited a more mesenchymal morphology and were found to be fibronectin positive. However, after cisplatin treatment, these clusters disappeared, and Ki67 expressing cells were observed all around the spheroids.

We recently showed that most HNSCC cell lines cultured as spheroids are more resistant to cetuximab and cisplatin treatment compared to HNSCC cells cultured as traditional two-dimensional cultures (2D) [6]. In the present study, we showed an increased effect of cetuximab (decreased cell proliferation) in spheroids with CAFs compared to spheroids solely comprising tumor cells, which was significant in both LK0917 and LK1108 cell lines. One explanation for this increased sensitivity to cetuximab could be the increase in EGFR expression, which is a target of cetuximab, that was seen in LK0917 and LK1108 tumor cells cultured with CAFs (Fig. 3). Others have shown that cetuximab also induces alterations in tumor cells by modifying genes and proteins involved in the extracellular matrix, which could be linked to CAFs [17].

Surprisingly, increased Ki67 positive cells were observed after three days of cisplatin treatment compared to controls in spheroids without CAFs in all analyzed cell lines. In our previous study, an increase in cell death was detected in two of the cell lines, LK0902 and LK0917, after seven days of cisplatin treatment with the same concentration as used in this study [6]. However, in LK0917 cells cultured with CAFs, a significant decrease in Ki67 expression was found compared to untreated tumor cell/CAF spheroids (Fig. 2). Ki67 is often used to estimate the proliferation rate but may not always reflect the proliferative state at the time of the assay, as Ki67 is expressed in all phases of the cell cycle, except G0 [18]. On the other hand, in this study, the levels of Ki67 positive cells correlated with the expression of EGFR, which may indicate that increased proliferation occurs early during cisplatin treatment with subsequent decreases in cell viability.

To further evaluate treatment response a TUNEL-assay was performed and in LK0902 and LK1108 spheroids only a minor increase in apoptosis was detected after treatment compared to untreated spheroids (Additional file 1: Figure S1 and Additional file 3: Figure S3). However, in LK0917 spheroids (with or without CAFs) an increase in apoptosis was found after cisplatin treatment and after cetuximab treatment in tumor cells/CAFs spheroids (Additional file 2: Figure S2). These results indicate that LK0917 spheroids are more sensitive to treatment but also due to the clusters of tumor cells with Ki67 positive cells and a more EMT phenotype (high fibronectin expression) probably have a higher metastatic potential than LK0902 and LK1108.

Expression of the growth receptor EGFR was investigated in untreated and treated cells (cisplatin and cetuximab) with and without CAFs. Increased expression of EGFR was observed in tumor cells cultured with CAFs, which correlated with increased positive Ki67 cells. To investigate the reason for the increase in EGFR and proliferation, further studies must analyses differences in gene expressions when tumor cells are co-cultured with CAFs or alone.

One of the aims for analyzing the EGFR and fibronectin expression was to study the growth pattern of tumor cells and CAFs in spheroids and to confirm the presence of CAFs. Interestingly, we found a similarity in the growth pattern of tumor cells and intensity of EGFR expression between 3-D cultures and tumor biopsies in all three samples (Additional file 6: Figure S6).

The phenotypic transition from epithelial to mesenchymal has been shown to have impact on CSC regulation and EMT-like cancer cells exhibit increased metastatic properties [19, 20]. In our previous study, HNSCC cell lines cultured as spheroids (3D) were compared to HNSCC cells cultured in 2D monolayers, and we found that tumor cells cultured in 3D acquire more a CSC-like phenotype [6]. Furthermore, clear differences were observed between cell lines regarding changes in EGFR and EMT-associated protein expression and drug efficacy [6]. Similar to other reports, we found increased expression of E-cadherin and decreased proliferation rate in all spheroids compared to cells grown in 2D [17]. Here, we compared mRNA expression of NANOG and SOX2 between tumor cells co-cultured with or without CAFs and found decreased mRNA expression of SOX2 in tumor cells co-cultured with CAFs from LK1108 (Fig. 4), but in the other two cell lines, a tendency to increased expression of SOX2 and NANOG was found.

Next, we investigated mRNA expression of genes involved in the epithelial-to-mesenchymal transition (EMT), and increased expression of FN1 and VIM was detected in LK0902 and LK0917 tumor cells co-cultured with CAFs. In LK1108 tumor cells, only FN1 was upregulated. These results indicate that CAFs induce an increased EMT-like phenotype in tumor cells in 3D after co-culturing with CAFs. However, these changes seem not to have any significant impact on cetuximab or cisplatin treatment sensitivity in our *in vit*ro model.

Our results show when using this unique in vitro model with tumor cells and CAFs from the same tumor that the treatment response and how the CAFs affect the phenotype varied a lot, which is also known for head and neck cancer patients. Therefore, it shows the importance in multi-drug screening investigations to use more than one in vitro model to get clinically useable results.

## Conclusions

Taken together, we have developed a unique tumor experimental in vitro model that consists of low passage HNSCC cells and CAFs from the same tumor. Our results indicate similarity in phenotype (growth pattern and EGFR expression) between tumor cell/CAF spheroids and the patient tumors. We further demonstrated that CAFs affect proliferation, EGFR expression and to some extent, the EMT and CSC phenotype of HNSCC tumor cells.

## Supplementary Information

**Additional file 1: Figure S1.** Ki67 expression and TUNEL-positivity in LK0902 cells grown in 3D ± CAFs after treatment with cisplatin and cetuximab. Immunohistochemical staining and TUNEL-staining of LK0902 tumor spheroids ± CAFs in response to treatment with cetuximab and cisplatin was measured in 5 days old tumor spheroids with the proliferation marker Ki67. Scale bar = 150 µm.

**Additional file 2: Figure S2.** Ki67 expression and TUNEL-positivity in LK0917 cells grown in 3D ± CAFs after treatment with cisplatin and cetuximab. Immunohistochemical staining and TUNEL-staining of LK0917 tumor spheroids ± CAFs in response to treatment with cetuximab and cisplatin was measured in 5 days old tumor spheroids with the proliferation marker Ki67. Clusters with tumor cells are indicated with arrows. Scale bar = 150 µm.

**Additional file 3: Figure S3.** Ki67 expression and TUNEL-positivity in LK1108 cells grown in 3D ± CAFs after treatment with cisplatin and cetuximab. Immunohistochemical staining and TUNEL-staining of LK1108 tumor spheroids ± CAFs in response to treatment with cetuximab and cisplatin was measured in 5 days old tumor spheroids with the proliferation marker Ki67. Scale bar = 150 µm.

**Additional file 4: Figure S4.** Cell viability of HNSCC cells grown in 3D after treatment with cisplatin. Cell viability upon treatment with cisplatin for 3 days was measured by MTS assay. Absorbance was measured at λ = 490 nm using an ELISA microplate reader. All measurements were performed in triplicate, and the data are shown as the mean ± SD; *p < 0.05 according to one-way ANOVA with Bonferroni adjustment.

**Additional file 5: Figure S5.** Fibronectin expression in HNSCC cells grown in 3D ± CAFs. Immunohistochemical staining of HNSCC tumor spheroids ± CAFs with fibronectin. (A, B) LK0902. (C, D) LK0917. (E, F) LK1108. Clusters with tumor cells are indicated with arrows. Scale bar = 150 µm.

**Additional file 6: Figure S6.** EGFR expression in HNSCC tumor biopsies and tumor spheroids. Expression of epidermal growth factor receptor (EGFR) was investigated by immunohistochemical staining. Scale bar = 150 µm.

## Data Availability

All data and material from the paper are available or can be requested from the corresponding author.

## Abbreviations

HNSCC: Head and neck squamous cell carcinoma
EMT: Epithelial–mesenchymal transition
CSCs: Cancer stem cells
EGFR: Epidermal growth factor receptor
CAFs: Cancer-associated fibroblasts
MACS: Magnetic activated cell sorting
