# Supplementary figures and images for: CAFs affect the proliferation and treatment response of head and neck cancer spheroids during co-culturing in a unique in vitro model
Source: Cancer Cell Int. 2020 Dec 22;20:599. doi: 10.1186/s12935-020-01718-6 (PMC7756959; doi:10.1186/s12935-020-01718-6)

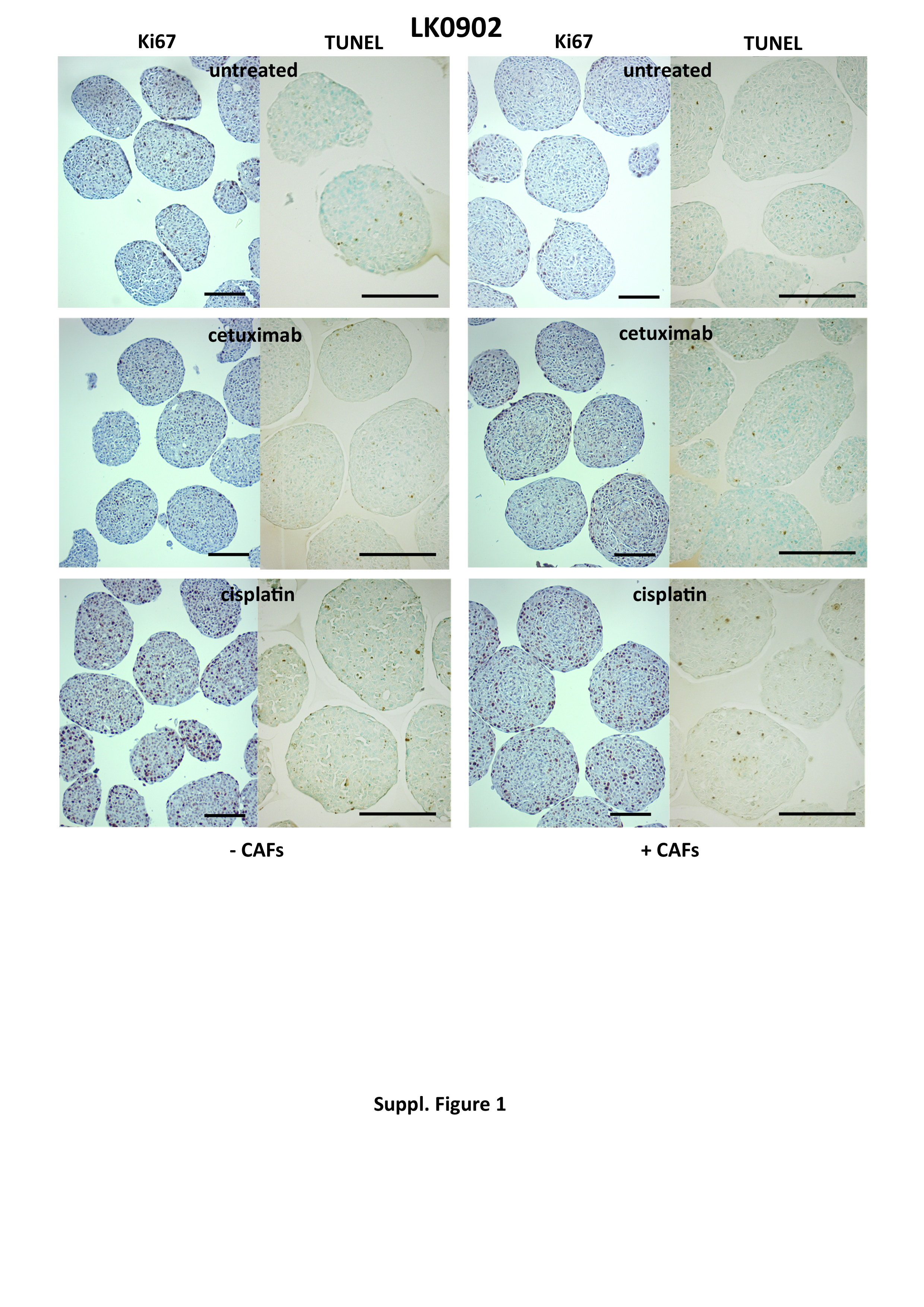

Supplement: Supplementary file 1 — Additional file 1: Figure S1. Ki67 expression and TUNEL-positivity in LK0902 cells grown in 3D ± CAFs after treatment with cisplatin and cetuximab. Immunohistochemical staining and TUNEL-staining of LK0902 tumor spheroids ± CAFs in response to treatment with cetuximab and cisplatin was measured in 5 days old tumor spheroids with the proliferation marker Ki67. Scale bar = 150 µm. [file 12935_2020_1718_MOESM1_ESM.tif]

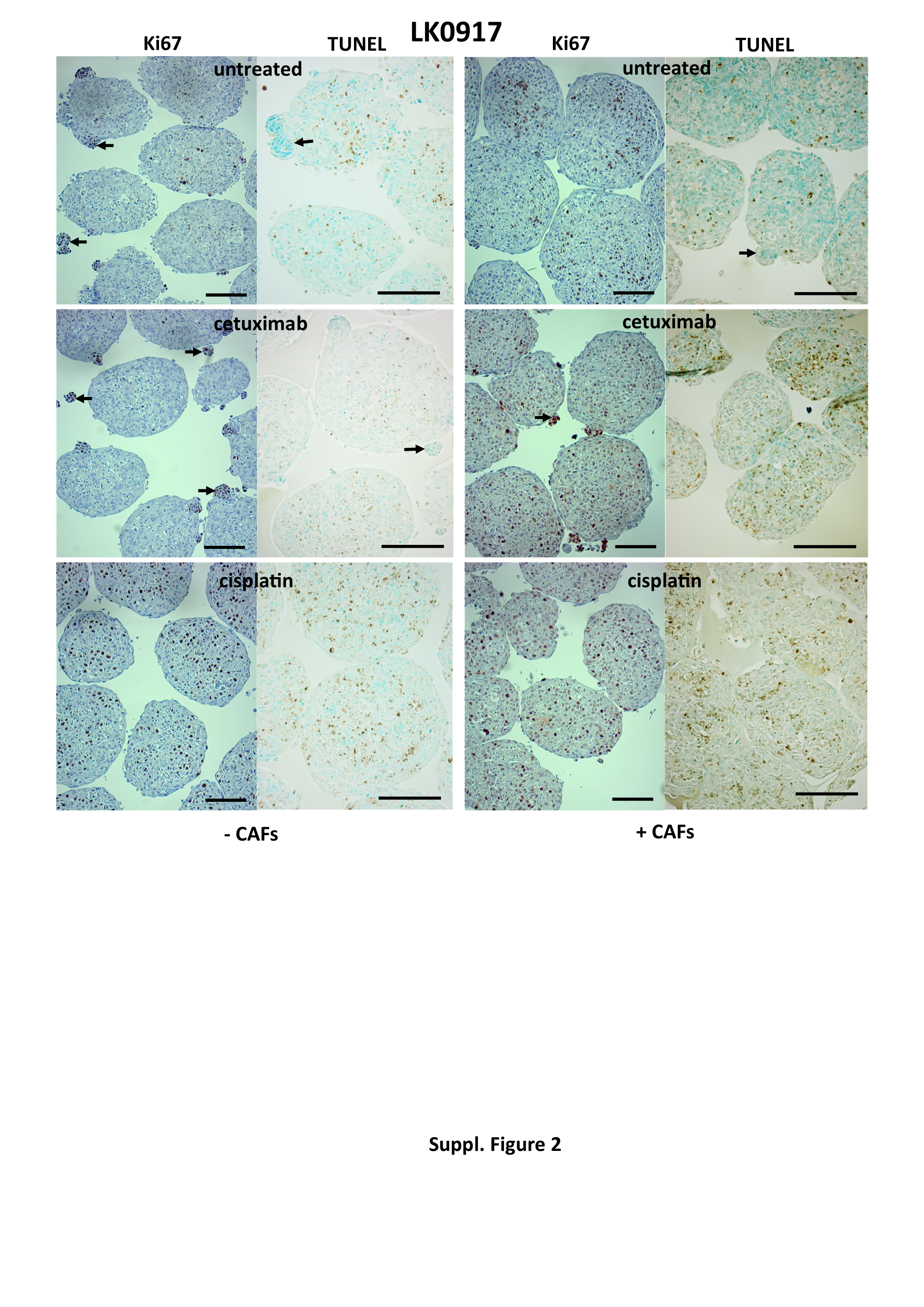

Supplement: Supplementary file 2 — Additional file 2: Figure S2. Ki67 expression and TUNEL-positivity in LK0917 cells grown in 3D ± CAFs after treatment with cisplatin and cetuximab. Immunohistochemical staining and TUNEL-staining of LK0917 tumor spheroids ± CAFs in response to treatment with cetuximab and cisplatin was measured in 5 days old tumor spheroids with the proliferation marker Ki67. Clusters with tumor cells are indicated with arrows. Scale bar = 150 µm. [file 12935_2020_1718_MOESM2_ESM.tif]

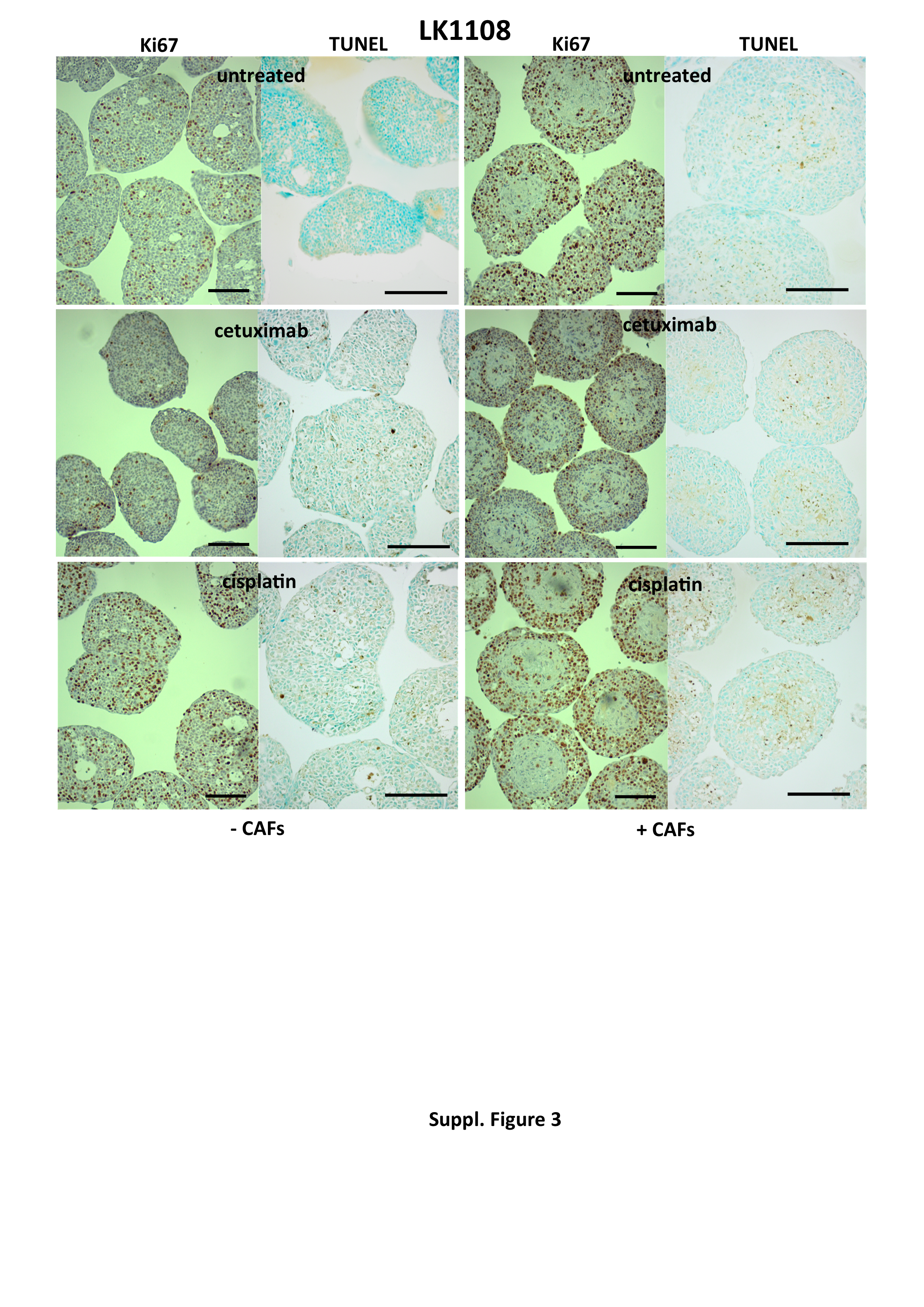

Supplement: Supplementary file 3 — Additional file 3: Figure S3. Ki67 expression and TUNEL-positivity in LK1108 cells grown in 3D ± CAFs after treatment with cisplatin and cetuximab. Immunohistochemical staining and TUNEL-staining of LK1108 tumor spheroids ± CAFs in response to treatment with cetuximab and cisplatin was measured in 5 days old tumor spheroids with the proliferation marker Ki67. Scale bar = 150 µm. [file 12935_2020_1718_MOESM3_ESM.tif]

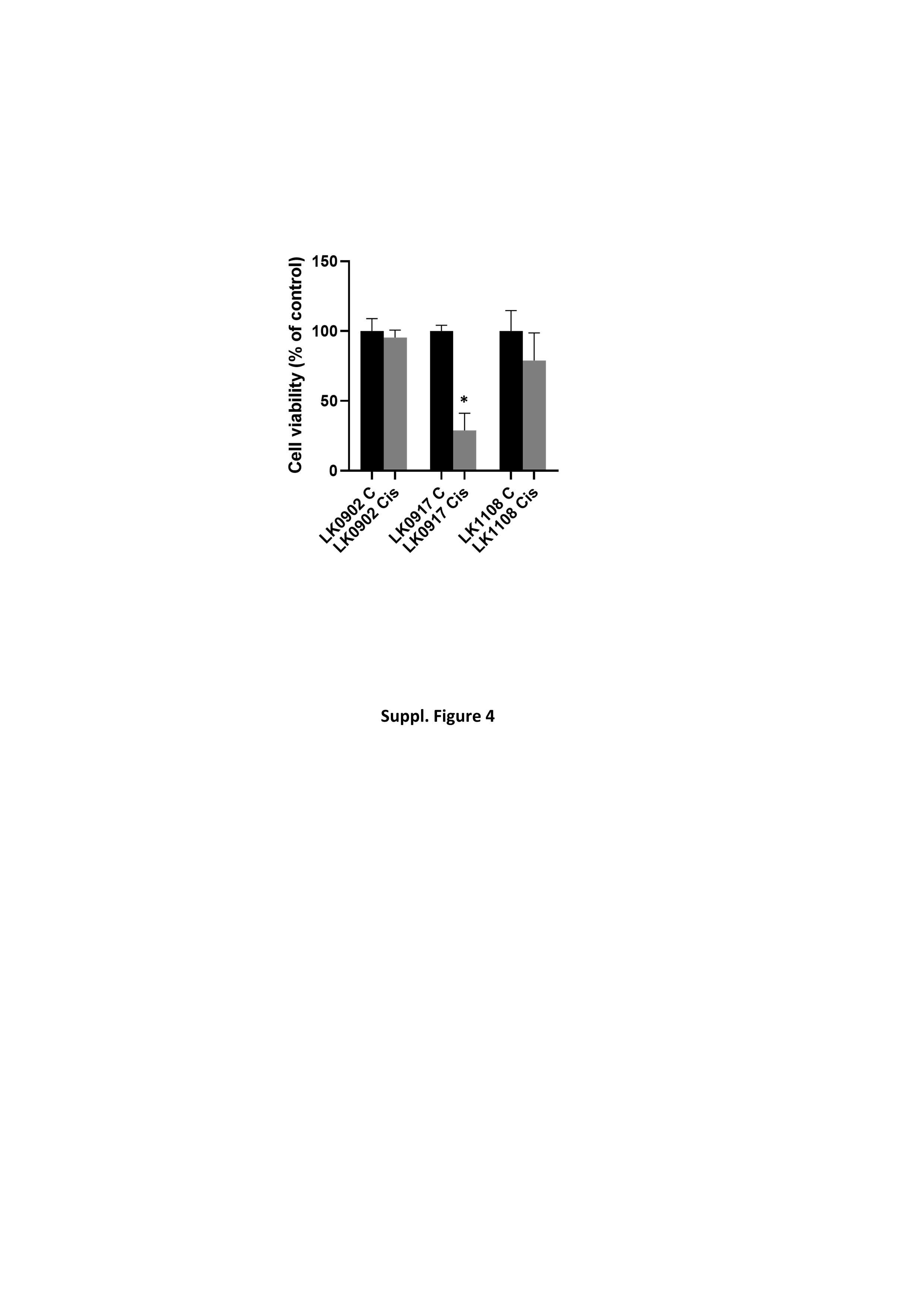

Supplement: Supplementary file 4 — Additional file 4: Figure S4. Cell viability of HNSCC cells grown in 3D after treatment with cisplatin. Cell viability upon treatment with cisplatin for 3 days was measured by MTS assay. Absorbance was measured at λ = 490 nm using an ELISA microplate reader. All measurements were performed in triplicate, and the data are shown as the mean ± SD; *p < 0.05 according to one-way ANOVA with Bonferroni adjustment. [file 12935_2020_1718_MOESM4_ESM.tif]

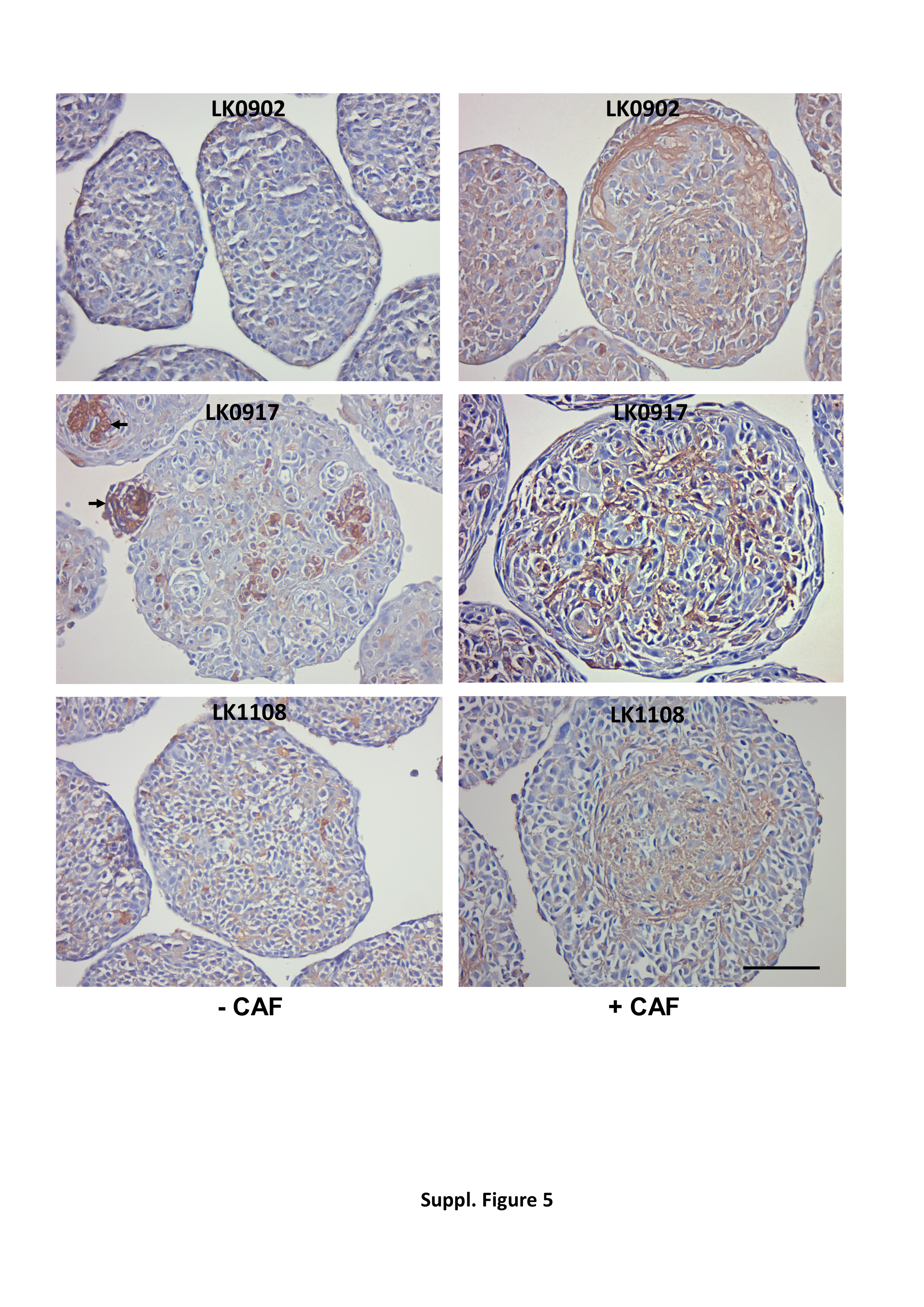

Supplement: Supplementary file 5 — Additional file 5: Figure S5. Fibronectin expression in HNSCC cells grown in 3D ± CAFs. Immunohistochemical staining of HNSCC tumor spheroids ± CAFs with fibronectin. (A, B) LK0902. (C, D) LK0917. (E, F) LK1108. Clusters with tumor cells are indicated with arrows. Scale bar = 150 µm. [file 12935_2020_1718_MOESM5_ESM.tif]

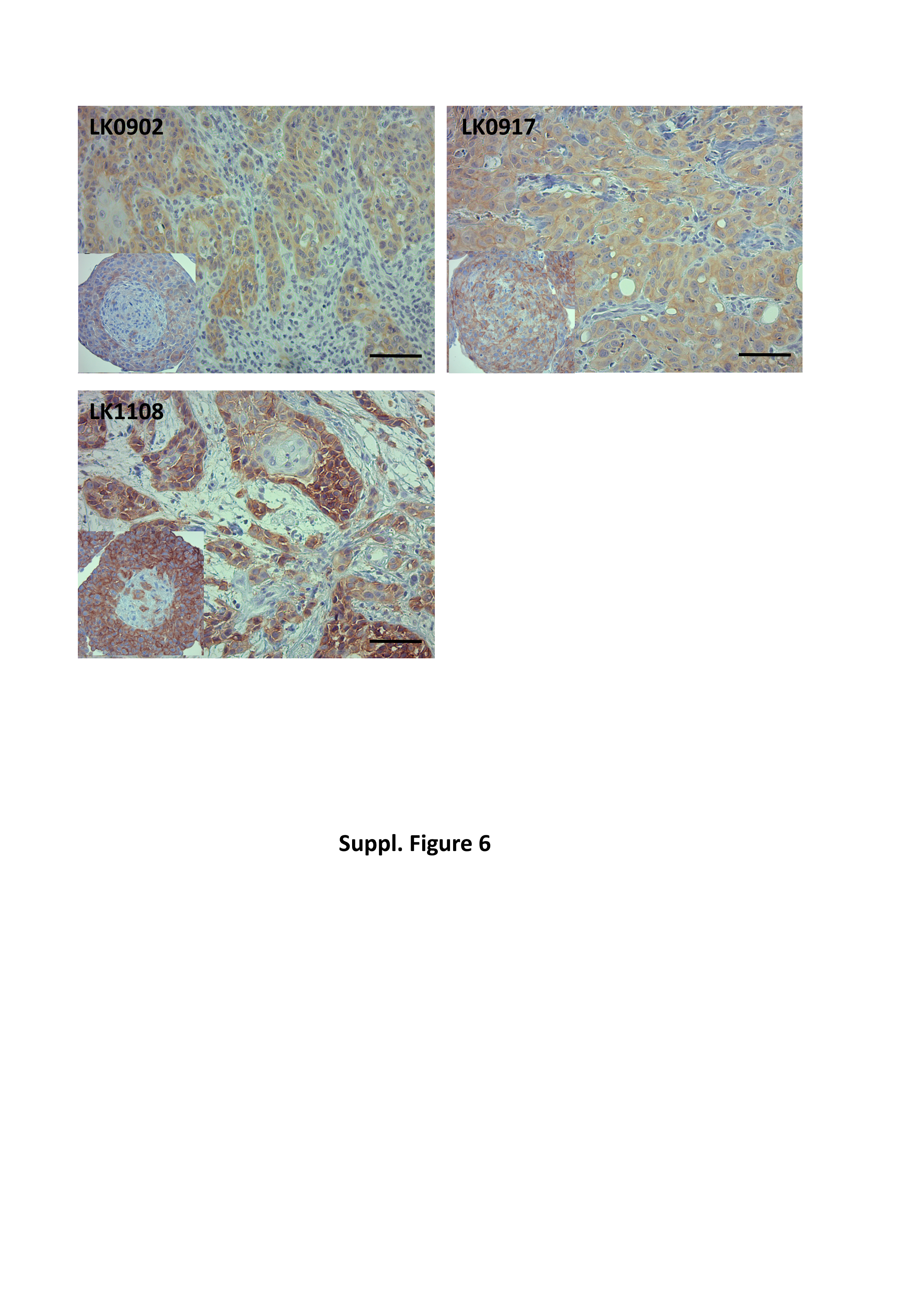

Supplement: Supplementary file 6 — Additional file 6: Figure S6. EGFR expression in HNSCC tumor biopsies and tumor spheroids. Expression of epidermal growth factor receptor (EGFR) was investigated by immunohistochemical staining. Scale bar = 150 µm. [file 12935_2020_1718_MOESM6_ESM.tif]
